# Electrode–Electrolyte Interactions in an Aqueous Aluminum–Carbon Rechargeable Battery System

**DOI:** 10.3390/nano11123235

**Published:** 2021-11-28

**Authors:** Jasmin Smajic, Amira Alazmi, Nimer Wehbe, Pedro M. F. J. Costa

**Affiliations:** 1 Physical Science and Engineering Division, King Abdullah University of Science and Technology (KAUST), Thuwal 23955-6900, Saudi Arabia; 2 Department of Chemistry, University Colleges at Nairiyah, University of Hafr Al-Batin, Hafr Al-Batin 39524, Saudi Arabia; amira.alazmi@uhb.edu.sa; 3 Core Labs, King Abdullah University of Science and Technology (KAUST), Thuwal 23955-6900, Saudi Arabia; nimer.wehbe@kaust.edu.sa

**Keywords:** carbon, graphene, aluminum, aqueous electrolyte, battery

## Abstract

Being environmentally friendly, safe and easy to handle, aqueous electrolytes are of particular interest for next-generation electrochemical energy storage devices. When coupled with an abundant, recyclable and low-cost electrode material such as aluminum, the promise of a green and economically sustainable battery system has extraordinary appeal. In this work, we study the interaction of an aqueous electrolyte with an aluminum plate anode and various graphitic cathodes. Upon establishing the boundary conditions for optimal electrolyte performance, we find that a mesoporous reduced graphene oxide powder constitutes a better cathode material option than graphite flakes.

## 1. Introduction

Lithium-ion batteries (LIBs) have developed into a reliable and high energy density solution for consumer electronics [1,2]. However, with the advent of electric vehicles and stationary electrochemical storage, there are mounting concerns related to their operational safety, cost and sustainability [3,4]. In fact, until recently, little consideration had been given to the environmental aspects of lithium extraction, which is known to cause freshwater shortages, chemical pollution and other adverse effects on ecosystems [5].

To address the above issues, much attention is being directed to alternative energy storage chemistries such as those based on Na, K, Mg, Zn and Al metals [6,7,8,9,10]. Of these, aluminum batteries are especially interesting. Al is considered a “green metal” as it is environmentally friendly, safe and easy to handle. Moreover, Al is the most abundant metal in the Earth′s crust, with an industrially mature approach for mineral extraction, processing and recycling. In particular, its recycling is economically attractive because of the overall low energy consumption (an estimated 5% of the energy needed to mine and process the ores). Today, 90% of Al used in transportation and construction, and around 75% of all that was mined is still in circulation [11,12,13,14]. Hence, aluminum is a prime resource to build a circular economy industry. Adding to this, and from an electrochemical perspective, Al can store three charge equivalents per mole, leading to theoretical gravimetric and volumetric capacities of 2980 mAh g^–1^ and 8046 mAh cm^–3^, respectively. These values are some of the highest known for battery materials [15,16].

For decades, the promise of Al batteries has been delayed by the lack of compatible electrolytes and cathodes. Aqueous electrolytes are the most desirable, due to safety and sustainability, but their adoption has largely failed as an oxide surface layer forms spontaneously on Al metal plates (anode) contacting water. In such circumstances, potentials that exceed the thermodynamic stability of water are required for the electrochemical cell to operate. Consequently, non-aqueous ionic liquids (IL) have been the electrolyte of choice [17,18]. In the most popular option, employing a deep eutectic melt based on AlCl_3_ and IL generates a solid electrolyte interface (SEI) that enables reversible stripping and plating of Al, mediated by the interconversion of AlCl_4_^-^ and Al_2_Cl_7_^-^ [19,20,21,22,23,24,25]. Regrettably, depending on their structural composition, these electrolytes can be toxic, or their biodegradability can be an issue. Additional challenges include their high cost, corrosivity and moisture sensitivity [25,26,27,28,29,30].

The interest in aqueous electrolytes for Al batteries was revived recently when Zhao et al. proposed an elegant step to make them compatible with Al anodes [31]. By first dipping the Al plate in an AlCl_3_-IL mixture, a protective coating is obtained that acts as an “artificial SEI” and prevents direct contact between the Al metal and aqueous electrolyte. Most importantly, the amount of AlCl_3_-IL needed is minimal. At present, there are few reports where the ex-situ “artificial SEI” is employed. In those, the choice of the cathode active material is rather limited to mostly metal oxides [31,32,33]. Despite showing promising capacity performance, the oxide-based cathodes suffer from inadequate stability and low capacity retention. Curiously, carbon cathodes, a popular choice in batteries, have not been explored for aqueous Al batteries integrating the aforementioned “artificial SEI” [34,35].

Here, in a bid to fabricate a low-cost, stable and environmentally friendly Al-battery, we build on top of Zhao′s conclusions and explore the stability of the “artificial SEI” approach in conjunction with a salt-water electrolyte and several graphitic carbon cathodes [29]. We find that the composition of the aluminum oxide surface layer (at the anode) is severely affected by the AlCl_3_-IL pretreatment and the electrolyte′s salt concentration. In addition, we observe that the graphitic carbon powders can have very interesting cathodic performance, provided their structure and chemistry are carefully tailored for this function.

## 2. Results and Discussion

The electrochemical window of aqueous electrolytes is notoriously narrow due to the H_2_ evolution that takes place at expanded potentials. This electrolyte degradation ought to be avoided if the longevity of the cell is to be maintained and catastrophic pressure buildup is to be averted. Assuming this, our first step was to test the electrolyte′s potential window of electrochemical stability. Popular amongst the group of “artificial SEI” Al anodes, the Al(OTF)_3_ salt was selected [31,32,33]. Circulating between Al (as-received state) and glassy carbon electrodes, the electrochemical response of the electrolyte was studied as a function of Al(OTF)_3_ concentration (Figure S1). Increasing the concentration of the aluminum salt effectively expanded the stability window of the electrolyte and suppressed the electrolysis of water. While a 1M solution offered a window of <0.5 V, for a 2M, the electrolyte was stable in the 0.2–2 V range. The 3M solution was stable for 2 V, whereas the more concentrated 5M expanded the upper interval limit past the 3 V mark. The observed trend is an expected consequence of the tight binding between Al^3+^ and its water solvation shell (ΔG_hydration_ = −4525 kJ mol^−1^) [36]. Increasing the concentration of the Al salt results in less free water molecules, thus stronger potentials are needed to induce the electrolyte decomposition. This makes the electrolyte more stable and, on a first view, the 5M solution our best option [37].

**Figure 1:**
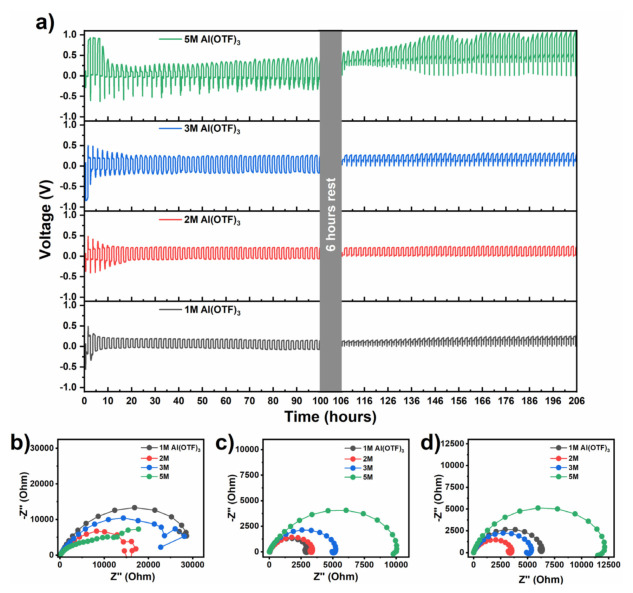
(**a**) Long−term stability of the electrolyte under a cyclic load of 0.01 mA cm^−2^, (**b**) electrochemical impedance spectroscopy (EIS) of the electrolyte before cycling, (**c**) EIS of the electrolyte after 100 h of charge−discharge cycles and (**d**) EIS of the electrolyte after 200 h of charge–discharge cycles.

Apart from the stability window, the concentration of the electrolyte must be chosen in accordance to its compatibility with other cell components. For this reason, a different story emerges when looking into the effects of repeated plating and stripping of the Al anode [38]. In Figure 1a, it is quite perceptible that the 5M electrolyte leads to an unreliable performance, with drifting potentials and an increase in polarization (from ~0.7 V at 20 h, to 1.0 V at 180 h), possibly due to increased viscosity and reduced ionic conductivity [39]. By contrast, the other electrolyte concentrations result in much more stable responses and smaller polarization values (0.35 V for 3M; 0.3 V for 2M; 0.25 V for 1M). Nevertheless, both the 1M and the 3M show sharp overshoot tails after a 6 h rest, instead of the desirable flat potentials. Such deviations are a fingerprint of inhibited charging/discharging processes. The 2M concentration, on the other hand, provides an extremely stable environment.

To understand the interfacial resistances contributing to the polarization and their dependence on the salt′s concentration, we probed the system at different time-points of the plating-stripping cycling (0 h, 100 h and 200 h), using electrochemical impedance spectroscopy (EIS). Immediately after the assembly of the cells (at 0 h), the EIS shows extremely high resistances (Figure 1b). At this stage, the interface between the Al anode and the electrolyte is very immature and the inductive loops at low frequencies are indicative of its modification through ion adsorption processes [39,40]. After 100 h of cycling (Figure 1c), lower resistances were seen. Both the 1M and 2M stand at around 3000 Ω, while the 3M and 5M show 5000 Ω and 10,000 Ω, respectively. By leaving the cells to rest for 6 h and cycling them for an additional 100 h (Figure 1d), a resistance of around 6500 Ω is measured for the 1M electrolyte, while the 2M and 3M have resistances of 3000 Ω and 5000 Ω, respectively. The 5M resistance increases to 12,000 Ω. From this, it is clear that the interface between the Al plate and the electrolyte is heavily dependent on the salt′s concentration and immersion time. If the concentration is too high or too low, the interface appears unstable as observed for the 1M and 5M electrolytes. On the other hand, both 2M and 3M appear stable and have low polarization values. In fact, the 2M has the lowest charge-transfer resistance and it is stable for more than 200 h of cycling (Figure 1a,d). Hereafter, in order to balance the interface stability, charge-transfer resistance and operational voltage window, a 2M electrolyte was used.

Even at the optimal salt concentration, the charge-transfer resistance across the SEI on the Al plates remains too high for practical application. Hence, we looked into modifying the metal′s surface by either polishing it with sandpaper (Emery, P1000), or exposing it to an AlCl_3_-IL electrolyte (to form an ex-situ “artificial SEI”) [31]. The analysis with EIS (Figure S2) shows that just polishing the foil reduces the resistance by one order of magnitude to 600 Ω (cf. Figure 1b,c). On the other hand, by just dipping the plate in the IL (as outlined by Zhao et al.), an even lower resistance of 250 Ω is obtained [28]. Clearly, mechanically removing the Al oxide film by polishing is not as good as the action of different organic and Cl-containing species in the AlCl_3_-IL mixture, at least to obtain a stable and sufficiently conductive SEI. Thus, and in agreement with previous studies, we used Al anodes with the artificial SEI for the rest of the experiments.

**Figure 2:**
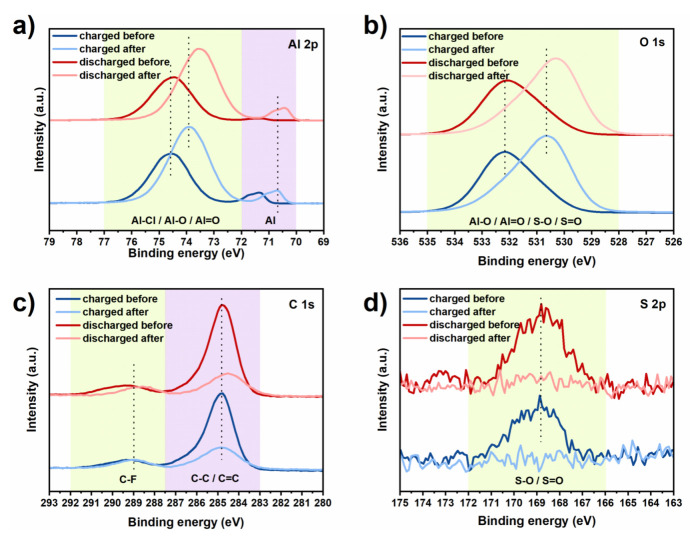
High−resolution X−ray photoelectron spectroscopy (XPS) spectra of the Al anode, in charged and discharged states, before and after the etching step: (**a**) Al 2p, (**b**) O 1s, (**c**) C 1s and (**d**) S 2p. The dashed lines are guides for the eyes. The legends of chemical bonds refer to the regions (outlined in green and purple colored background) where they are commonly found.

Next, X-ray photoelectron spectroscopy (XPS) was used to examine the chemical composition of the electrochemically–cycled Al anode (after 100 cycles) that had the “artificial SEI” coating. A depth profile assessment was rendered possible by etching the Al plates with a high-energy Ar beam. Besides the survey XPS spectra (Figure S3), high-resolution analysis of the Al 2p, O 1s, C 1s and S 2p photoelectrons was performed before and after the etching procedure, with the plates in either charged or discharged states (Figure 2). All Al 2p spectra (Figure 2a) show two major features: (1) a smaller unsymmetrical signal, in the 72–70 eV range, that is attributed to spin-orbit splitting in metallic aluminum, and 2) a dominant symmetrical peak, in the 77–72 eV range. The latter can arise from both the Al-Cl component of the SEI and the Al–O/Al=O bonds from the native oxide and solvated Al^3+^ ions [41,42]. This explains the different binding energies (BE) of the peaks, before and after etching. For instance, with increased depth, the dominant peak (77–72 eV range) shifts to lower binding energy (BE) due to a decrease in Al–Cl presence and a relative increase in the contribution of Al–O/Al=O components [41]. Similarly, the BE of the metallic aluminum (72–70 eV) is reduced post-etching, explained by the differences between surface and bulk Al atoms [43]. Curiously, the intensity of the metallic aluminum peaks, in the charged state and before etching, resembles that in discharged state after etching. A possible explanation is the partial electrodeposition of Al on top of the ex-situ “artificial SEI” layer. This is opposite to the discharged state, wherein the metallic aluminum peak before etching has a significantly lower intensity than the post-etching one. We understand it as a consequence of the solvated Al ions migrating towards the anode during charging and diffusing in the opposite direction (i.e., away from the anode) during discharge. This is further confirmed by the higher BE of the charged state after etching.

The Al 2p analysis was complemented by the O 1s study (Figure 2b). Again, a BE shift was observed as a function of depth. The peaks are asymmetric and can originate from the electrolyte (S–O/S=O present in CF_3_SO_3_^−^), the Al^3+^ solvation shell (Al–O) and the native aluminum oxide (Al–O/Al=O). The etching step resulted in a ~2 eV shift to lower BE. This is explained by the elimination of the IL component and subsequent exposure of the aluminum oxide, in agreement with the Al 2p findings. After etching, the visibly higher BE of the charged state is explained by an increased presence of solvated Al ions. Not surprisingly, the C 1s and S 2p spectra are less informative (Figure 2c,d, respectively). The C 1s spectrum consists of two main peaks. The smaller one, in the 291–288 eV range, is attributed to the C–F from the electrolyte and has constant intensity. The dominant peak, in the 287–283 eV range, originates from the organic component of the ionic liquid, [EMIm] (i.e., C–C/C=C), that integrates the AlCl_3_-IL pretreatment [44]. This is also the region of adventitious C (284.8 eV). Upon etching, its intensity decreases significantly, as expected. Likewise, the S 2p peaks (from the SO_3_^-^ of the Al(OTF)_3_ electrolyte) vanish after etching. Following the peak shift analysis, the elements identified with XPS were quantified, before and after etching (Table 1).

On average, the Al and O presence increases by ~10 at%, while that of C decreases by ~15 at%. Albeit present in small quantities before etching, the concentration of F and Cl decays further, indicating limited but stable adsorption on the anode. On the other hand, the little S that was identified, decreases below the detection limit after etching. Taken together, the native aluminum oxide layer in the anode is clearly affected by the IL pretreatment. However, the IL coating is also modified by the electrolyte. An optical and electron microscopy analysis of the surface (Figure S4Figure S5) identified pits, indicating that the aforementioned SEI does not inhibit the stripping of Al^3+^ ions.

**Figure 3:**
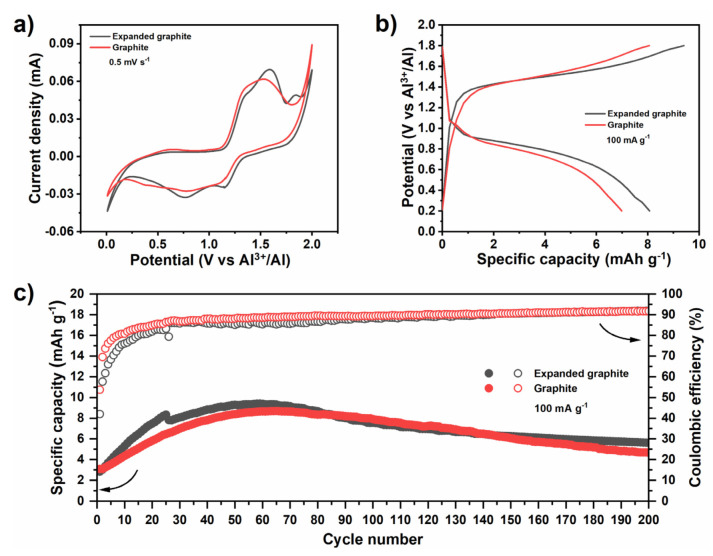
Electrochemical performance of the graphite and expanded graphite cathode: (**a**) cyclic voltammetry, (**b**) charge−discharge voltage profiles and (**c**) cycling stability with Coulombic efficiency.

After identifying the characteristics of the cycled Al anode, we assessed the available repertoire of cathodes for aqueous Al batteries. Confronted with the predominance of metal oxides, we looked for possible alternatives and tested two of the most common carbon electrode materials: graphite and expanded graphite (i.e., processed expandable graphite). As seen in Figure 3a, their cyclic voltammetry exhibit broad redox peaks that can be attributed to the intercalation of ions into the layered carbons.

**Figure 4:**
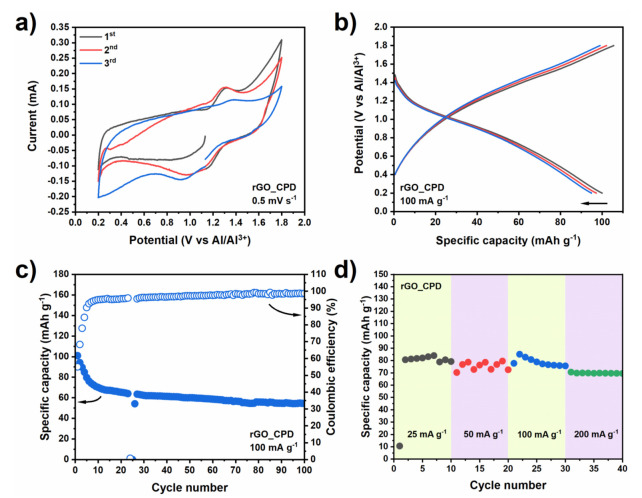
Electrochemical performance of the supercritically−dried reduced graphene oxide (rGO_CPD): (**a**) cyclic voltammetry, (**b**) charge−discharge voltage profiles, (**c**) cycling stability with Coulombic efficiency and (**d**) rate capability.

The voltage curves (Figure 3b) show plateaus centered at 0.9 V and 1.4 V, for discharge and charge, respectively. These agree well with the cyclic voltammetry. The capacity, however, peaks at around 10 mAh g^−1^ (Figure 3c), declining by ~50% after 200 cycles. Assuming that the Al^3+^ (a strong Lewis acid) remains solvated throughout, this behavior is a likely consequence of the large size of the intercalant (0.38 nm) [45]. This hypothesis is also supported by a slightly higher capacity of expanded graphite, due to its larger interlayer distance. Carbons are a versatile family of materials. Besides a number of allotropes, it is possible to tailor their structure and surface chemistry [46]. In fact, depending on the process selected, powders derived from graphite can be modified to accommodate large ions. Previously, we demonstrated the importance of the drying method as we tuned the porosity of reduced graphene oxide (rGO) to optimize its performance for batteries and supercapacitors [19,47]. Supercritical drying methods yield rGO powders with a moderate surface area and an average pore size of 20 nm that could accommodate the large size of solvated Al^3+^ ions through mostly pseudocapacitive charge storage [19,47]. In these circumstances, allying the chemical stability of graphitic carbon powders to the mesoporosity of the rGOs could be advantageous when exploring alternative cathode materials for aqueous Al batteries (Figure 4).

As seen in Figure 4, the supercritically-dried rGO (rGO_CPD) exhibits vastly superior performance than both graphite and expanded graphite. Its cyclic voltammogram (Figure 4a) shows broad at peaks 1.0 V and 1.3 V for discharge and charge, respectively. These potential values are similar to those referred to above, for the graphite and expanded graphite. The intensity of the rGO_CPD peaks is relatively lower but the area enclosed by the cyclic voltammogram curves is larger, indicating an increased pseudocapacitive contribution to the charge-storage mechanism [48,49]. The voltage curves (Figure 4b) show sloping profiles, instead of plateaus, in agreement with the pseudocapacitive character of the electrode and justifies the remarkable increase in capacity (by one order of magnitude), with the initial capacity at ~100 mAh g^−1^. Furthermore, the rGO_CPD′s cycling stability and the coulombic efficiency are also superior (Figure 4c), with 77% of capacity retention between 10th and 100th cycles at a constant coulombic efficiency of ~98%. Finally, its rate capability (Figure 4d) yields similar capacities for different current densities, confirming the resilience of the rGO_CPD cathode. Such a response speaks to the stability and efficiency of this system. It also demonstrates that “opening up” the graphitic structure (by increasing the surface area and inducing mesoporosity), successfully overcomes the inherent limitations of the large ion size. Ultimately, the ~20 nm mesopores network of the rGO_CPD enables a better wetting of the cathode mass as well as facilitating the access of the Al ions to redox-active sites [19,47].

## 3. Conclusions

A modified Al anode, a reduced graphene oxide cathode and a 2M Al(OTF)_3_ electrolyte can be used to form an eco-friendly and inherently safe rechargeable battery. We find that the SEI layer on the Al anode is dependent on both the pretreatment method and the electrolyte. Furthermore, increasing the pseudocapacitive component of the carbon cathode is beneficial in improving the electrochemical performance of an aqueous Al battery.

## 4. Experimental

### 4.1. Materials Synthesis

Graphite (Alfa Aesar; 99.99% purity; 44 μm average flake size) and expanded graphite were used as-received [50]. To prepare the reduced graphene oxide (rGO), we followed a procedure described elsewhere [51]. Briefly, graphite powder (99%, crystalline, 325 mesh, Alfa Aesar) was oxidized and exfoliated using the improved Hummers′ method. Following this, the graphene oxide (GO) powder was dried with supercritical CO_2_ for 24 h, and hydrothermally reduced, at 180 °C for 24 h. Upon collection, the rGO product was dried under supercritical conditions (for 24 h).

The aqueous electrolyte was prepared by mixing the salt aluminum trifluoromethanesulfonate Al(OTF)_3_ (Alfa Aesar) with appropriate volumes of deionized water to obtain various concentrations, 1M, 2M, 3M and 5M. To prepare the ionic liquid electrolyte, 1-ethyl-3-methyl imidazolium chloride [EMIm]Cl was first annealed in a tube furnace, in vacuo, at 70 °C and for 16 h, to remove residual water. This powder was then introduced into an Ar-filled glove box (MBraun LabStar, <0.5 ppm O_2_, <0.5 ppm H_2_O), always avoiding exposure to air. The anhydrous AlCl_3_ powder was used as received from the vendor (Alfa Aesar). With these two components, the AlCl_3_: [EMIm]Cl = 1.3 (mol mol^−1^) electrolyte was prepared by slowly adding the AlCl_3_ to [EMIm]Cl, inside the glove box. Mixing of the two powders produced a clear light-yellow liquid through an exothermic reaction. Finally, the electrolyte obtained was stirred for 30 min and left to stand.

### 4.2. Materials Characterization

Optical microscopy was performed on a Zeiss STEMI 2000-C. The X-ray photoelectron spectroscopy (XPS) characterization was undertaken on a Kratos Axis Ultra, equipped with a monochromatic Al Kα X-ray source (hν = 1486.6 eV) and operated at a power of 150 W under ultra high-vacuum conditions (10^–9^ mbar). The etching process was performed using argon cluster beam Ar500+, operated at 5 keV. The raster size of the etched area was about 4 mm × 4 mm and the etching was done until S could not be detected (~30–50 nm). The spectra were collected from the middle of the etched area, in order to avoid the edge effect. Scanning electron microscopy (SEM) was done on an FEI Nova Nano operated at 5 kV.

### 4.3. Electrochemical Measurements

The electrochemical studies were undertaken with two potentiostats, a Bio-Logic VMP3 and a BCS-800, in a 2023 coin cell configuration. Electrochemical impedance spectroscopy (EIS) was carried out from 10 mHz to 100 kHz with an amplitude of 10 mV. The cells were assembled using the carbon powders and an Al foil (99.999%, 0.25 mm, Sigma-Aldrich) as the active electrode materials (cathode and anode, respectively). The cathode was prepared by sandwiching the carbons (~1 mg cm^−2^) between a glass fiber separator and a current collector. No binders were used. Carbon paper was the current collector of choice for the electrochemical performance assessment. In all experiments, 100 µL of aqueous electrolyte was used for each cell.

## Figures and Tables

**Table 1:** Elemental analysis (at%) of the Al anode′s surface, as calculated from XPS measurements.

	Al	O	C	S	F	Cl
Charged before	25.2	45.8	25.0	0.4	1.6	1.9
Charged after	34.3	52.8	11.4	n/a	0.8	0.6
Discharged before	21.7	45.5	29.7	0.4	1.3	0.3
Discharged after	33.7	52.4	11.4	n/a	0.6	n/a

## Data Availability

The raw/processed data required to reproduce these findings cannot be shared at this time as the data also forms part of an ongoing study.

## Supplementary Materials

The following are available online at www.mdpi.com/article/10.3390/nano11123235/s1, Figure S1: Electrochemical voltage window of stability for the Al(OTF)_3_ electrolyte at different concentrations (1M to 5M); Figure S2: EIS of the Al anode with different pretreatment methods (all taken before cycling, i.e. 0 h); Figure S3: Survey XPS spectra of the charged and discharged Al anode’s surface before and after etching. The dashed lines are guides for the eyes; Figure S4: Optical microscopy images of the aluminum anode surface after electrochemical cycling; Figure S5: (a–c) SEM images of the aluminum anode surface after electrochemical cycling.
