# Supplementary material for: Electrode–Electrolyte Interactions in an Aqueous Aluminum–Carbon Rechargeable Battery System
Source: Nanomaterials (Basel). 2021 Nov 28;11(12):3235. doi: 10.3390/nano11123235 (PMC8704015; doi:10.3390/nano11123235)
Supplement: Supplementary file 1 [file nanomaterials-11-03235-s001.zip › nanomaterials-1472988-supplementary.pdf]

nanomaterials
MDPI

SUPPORTING INFORMATION

Electrode–Electrolyte Interactions in an Aqueous Aluminum–Carbon Rechargeable Battery System

Jasmin Smajic [1,*], Amira Alazmi [2], Nimer Wehbe [3] and Pedro M. F. J. Costa [1,*]

1 Physical Science and Engineering Division, King Abdullah University of Science and Technology (KAUST), Thuwal 23955-6900, Saudi Arabia;
2 Department of Chemistry, University Colleges at Nairiyah, University of Hafr Al-Batin, Hafr Al-Batin 39524, Saudi Arabia; amira.alazmi@uhb.edu.sa
3 Core Labs, King Abdullah University of Science and Technology (KAUST), Thuwal 23955-6900, Saudi Arabia; nimer.wehbe@kaust.edu.sa
* Correspondence: jasmin.smajic@kaust.edu.sa (J.S.); pedro.dacosta@kaust.edu.sa (P.M.F.J.C.)

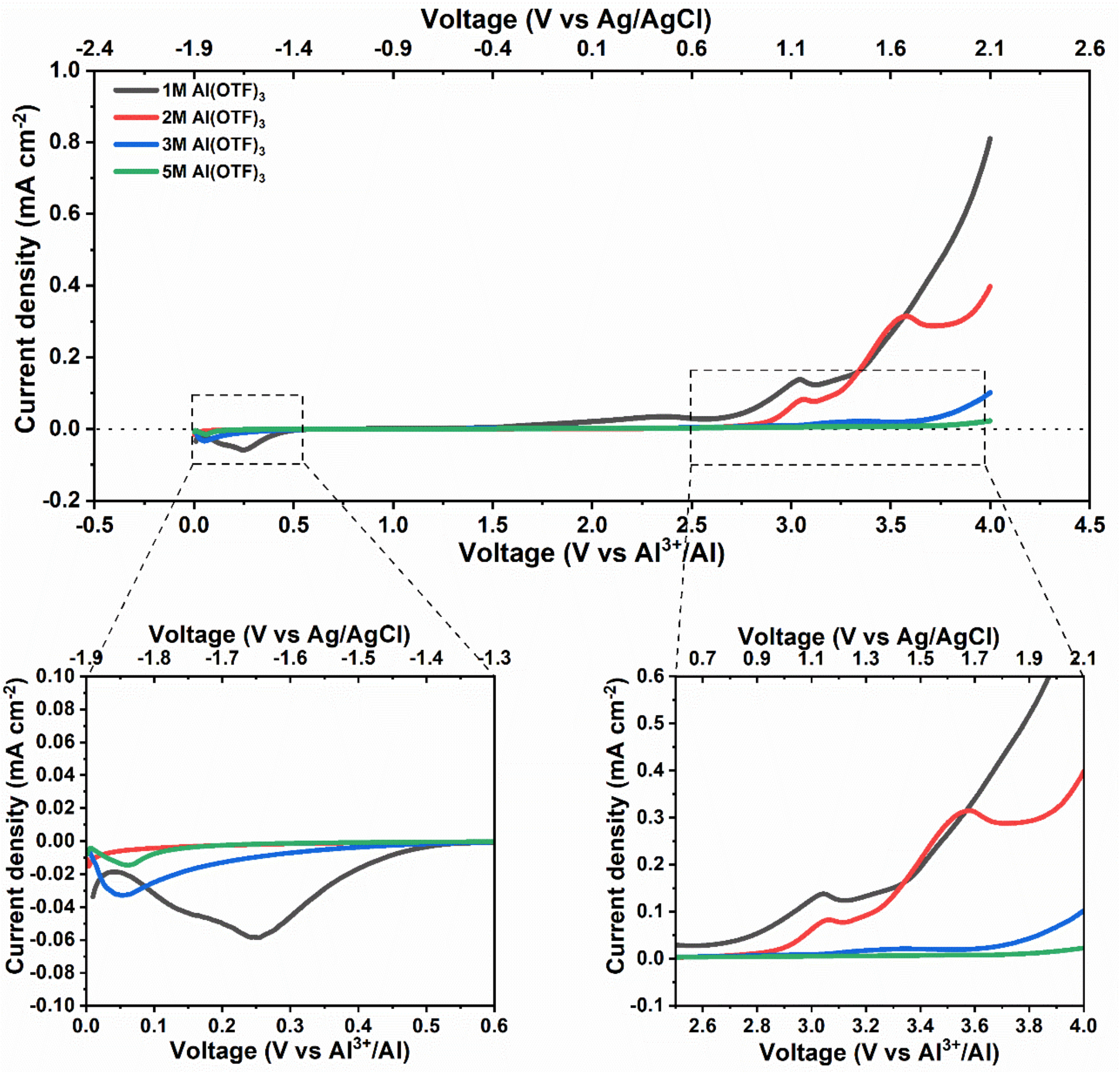

Figure S1. Electrochemical voltage window of stability for the Al(OTF)3 electrolyte at different concentrations (1M to 5M).

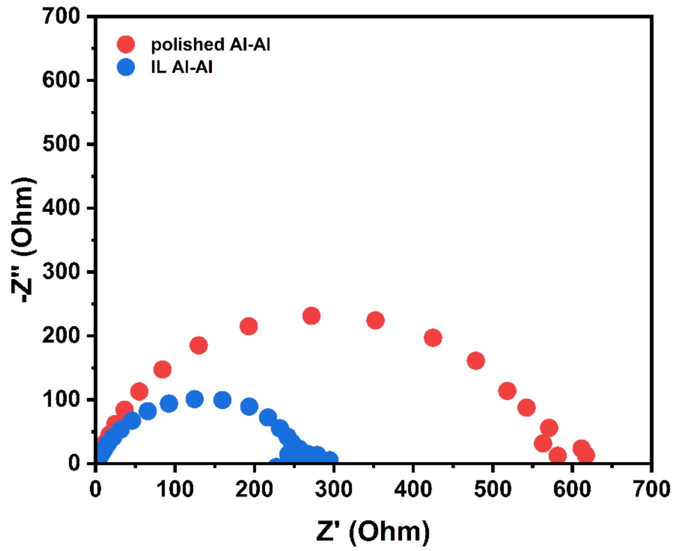

Figure S2. EIS of the Al anode with different pretreatment methods (all taken before cycling, i.e. 0 h).

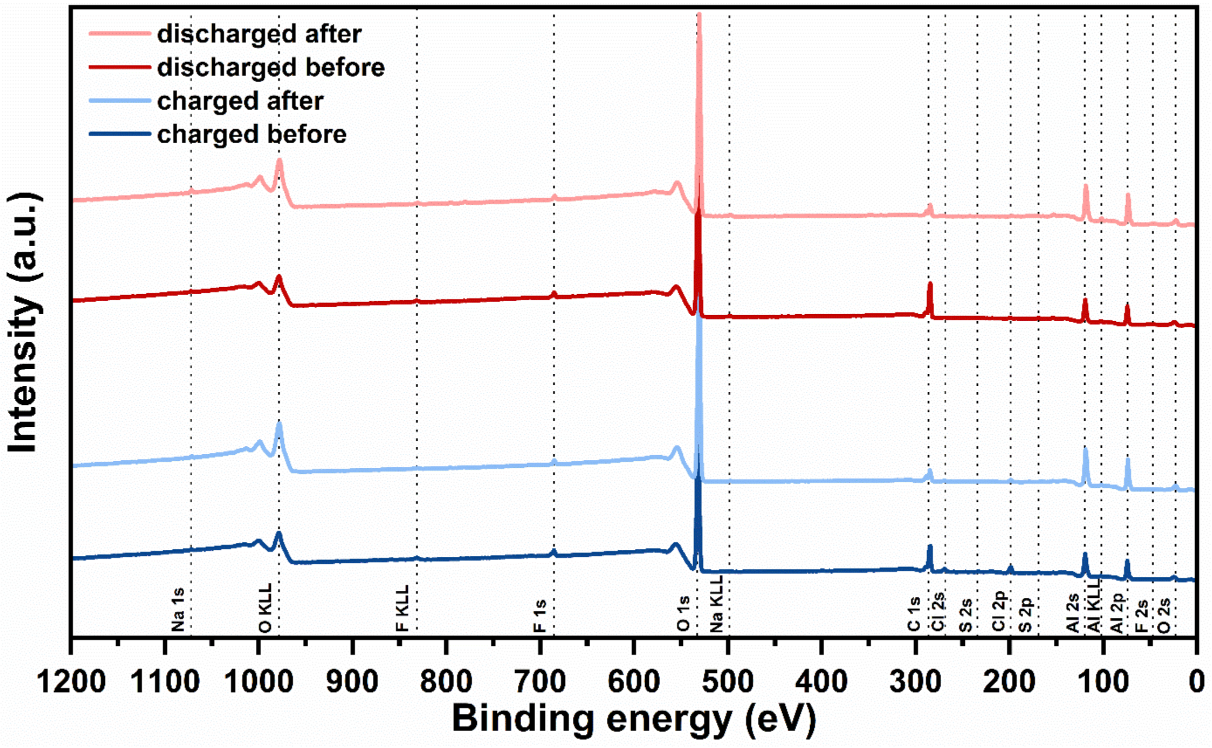

Figure S3. Survey XPS spectra of the charged and discharged Al anode's surface before and after etching. The dashed lines are guides for the eyes.

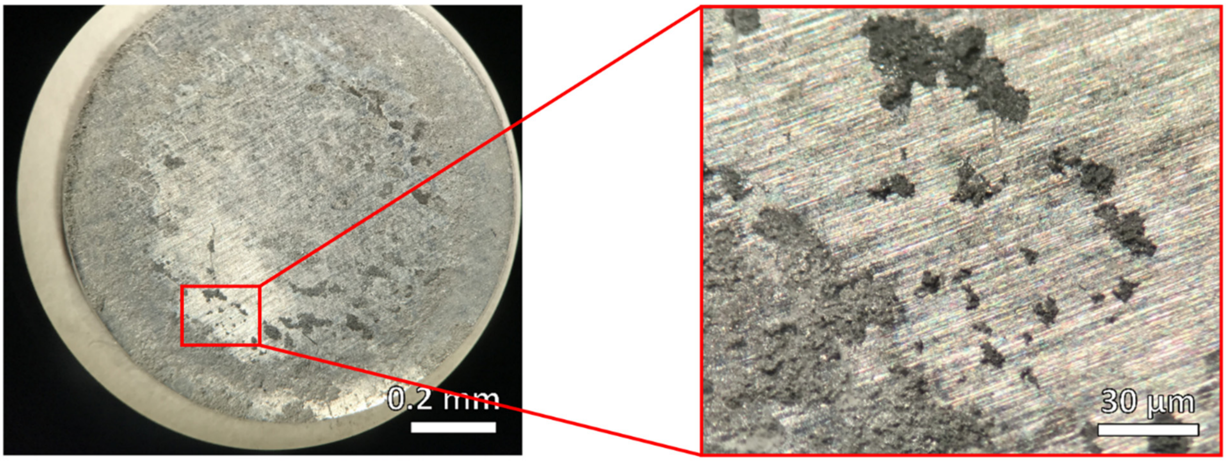

Figure S4. Optical microscopy images of the aluminum anode surface after electrochemical cycling.

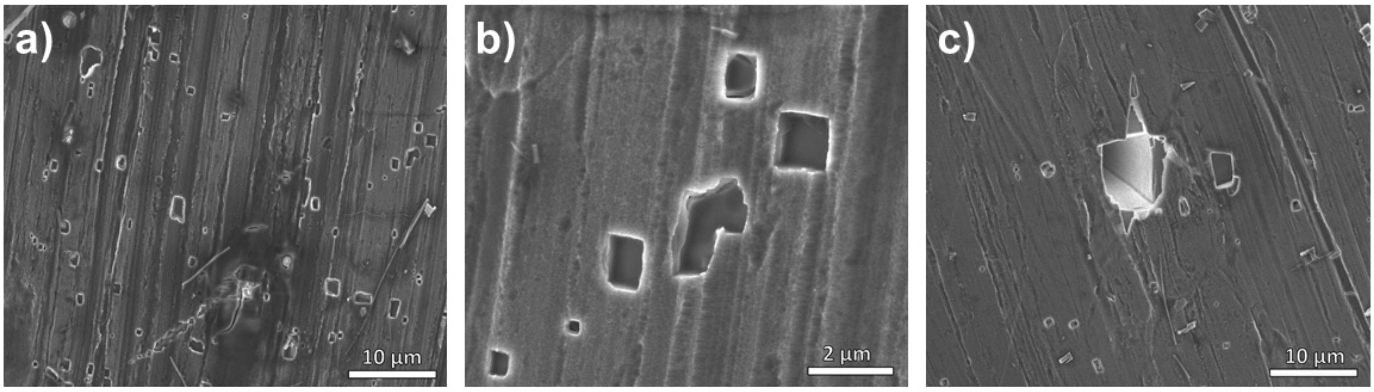

Figure S5. (**a–c**) SEM images of the aluminum anode surface after electrochemical cycling.
